# Rapid virulence prediction and identification of Newcastle disease virus genotypes using third-generation sequencing

**DOI:** 10.1186/s12985-018-1077-5

**Published:** 2018-11-22

**Authors:** Salman L. Butt, Tonya L. Taylor, Jeremy D. Volkening, Kiril M. Dimitrov, Dawn Williams-Coplin, Kevin K. Lahmers, Patti J. Miller, Asif M. Rana, David L. Suarez, Claudio L. Afonso, James B. Stanton

**Affiliations:** 1 Southeast Poultry Research Laboratory, US National Poultry Research Center, Agricultural Research Service, USDA, 934 College Station Road, Athens, GA 30605 USA; 2 Department of Pathology, College of Veterinary Medicine, University of Georgia, Athens, GA 30602 USA; 3 BASE2BIO, Oshkosh, WI USA; 4 Department of Biomedical Sciences & Pathobiology,VA-MD College of Veterinary Medicine, Virginia Tech, Blacksburg, VA USA; 5 Hivet Animal Health Business, 667-P, Johar Town, Lahore, Pakistan; 6 Department of Population Health, College of Veterinary Medicine, 953 College Station Road, Athens, GA 30602 USA

**Keywords:** Newcastle disease virus, RNA, Genotype, Nanopore sequencing, Rapid sequencing, MinION, NGS

## Abstract

**Background:**

Newcastle disease (ND) outbreaks are global challenges to the poultry industry. Effective management requires rapid identification and virulence prediction of the circulating Newcastle disease viruses (NDV), the causative agent of ND. However, these diagnostics are hindered by the genetic diversity and rapid evolution of NDVs.

**Methods:**

An amplicon sequencing (AmpSeq) workflow for virulence and genotype prediction of NDV samples using a third-generation, real-time DNA sequencing platform is described here. 1D MinION sequencing of barcoded NDV amplicons was performed using 33 egg-grown isolates, (15 NDV genotypes), and 15 clinical swab samples collected from field outbreaks. Assembly-based data analysis was performed in a customized, Galaxy-based AmpSeq workflow. MinION-based results were compared to previously published sequences and to sequences obtained using a previously published Illumina MiSeq workflow.

**Results:**

For all egg-grown isolates, NDV was detected and virulence and genotype were accurately predicted. For clinical samples, NDV was detected in ten of eleven NDV samples. Six of the clinical samples contained two mixed genotypes as determined by MiSeq, of which the MinION method detected both genotypes in four samples. Additionally, testing a dilution series of one NDV isolate resulted in NDV detection in a dilution as low as 10^1^ 50% egg infectious dose per milliliter. This was accomplished in as little as 7 min of sequencing time, with a 98.37% sequence identity compared to the expected consensus obtained by MiSeq.

**Conclusion:**

The depth of sequencing, fast sequencing capabilities, accuracy of the consensus sequences, and the low cost of multiplexing allowed for effective virulence prediction and genotype identification of NDVs currently circulating worldwide. The sensitivity of this protocol was preliminary tested using only one genotype. After more extensive evaluation of the sensitivity and specificity, this protocol will likely be applicable to the detection and characterization of NDV.

**Electronic supplementary material:**

The online version of this article (10.1186/s12985-018-1077-5) contains supplementary material, which is available to authorized users.

## Background

Newcastle disease (ND) is one of the most important infectious diseases of poultry and is a major economic burden to the global poultry industry. Virulent strains of avian paramyxovirus 1 (APMV-1), commonly known as Newcastle disease virus (NDV) [1], are the cause of ND and have been recently reclassified as avian avulavirus-1 (AAvV-1) [2]. Newcastle disease viruses are a highly diverse group of viruses with two distinct classes, 19 accepted genotypes and a wide host range including domestic and wild bird species. In addition to the genotypic diversity of NDVs, these viruses are also diverse in their virulence. This includes low virulent viruses, whose replication is limited to the respiratory and digestive tracts and typically cause clinically inapparent infections, to highly virulent viruses that cause acute disease with high mortality rates [1, 3]. The global spread, constant evolution, varying virulence, and the wide host range of NDV are challenges to the control of ND [4].

Effective control of ND is dependent on specific diagnostic testing, which is typically oriented towards detection, genotyping, or prediction of virulence. Virulence of NDV is best assayed through in vivo pathogenicity studies [5], but due to the cost and time constraints associated with such methods, reverse transcriptase-quantitative PCR (RT-qPCR) and sequencing of the F gene cleavage site are used to predict NDV virulence [6, 7]. Genotyping of NDV is commonly achieved through sequencing of the coding sequence of the fusion gene [8], which also allows for prediction of virulence. Preliminary genotyping can be accomplished through partial fusion gene sequencing (i.e., variable region) [9]. For detection of NDV, PCR assays avoid the highly variable fusion gene and instead target more conservative regions of the genome (i.e., matrix and polymerase genes) [10–14]; however, while this increases the applicability of these assays across genotypes, these assays lack applicability for virulence and genotypic determination. For example, while fusion-based assays can be used for detection [10, 15], the variability of this region, which makes it useful for genotyping, hinders the universal applicability of any single primer set [11, 12] and often requires screening samples with a different PCR assay prior to pathotyping [15]. Furthermore, for most current methods, detection, genotyping, and virulence prediction rely on Sanger sequencing; thus, they lack multiplexing capability and have limited sequencing depth, which complicates detection of mixed infections. In summary, there is a need to develop a method that will sensitively and rapidly detect NDV from multiple genotypes, while also providing genotype and virulence predictions.

Rapid advances in nucleic acid sequencing have led to different sequencing platforms [16, 17] being widely applied for identification of novel viruses [18], whole genome sequencing [19], transcriptomics, and metagenomics [20, 21]. However, high capital investments and relatively long turnaround times limit the widespread use of these next-generation sequencing (NGS) platforms, especially in developing countries [22]. Recent improvements in third-generation sequencing, including those introduced by Oxford Nanopore Technologies (ONT) [23], increase the utility of high-throughput sequencing as a useful tool for surveillance and pathogen characterization [24]. Among the transformative advantages of ONT’s sequencing technology are the ability to perform real-time sequence analysis with a short turnaround time [25], the portability of the MinION device, the low startup cost compared to other high-throughput platforms, and the ability to sequence up to several thousand bases from individual RNA or DNA molecules. The MinION device has been successfully used to evaluate antibiotic resistance genes from several bacterial species [26, 27], obtain complete viral genome sequences of an influenza virus [28] and Ebola virus [29], and detect partial viral genome sequences (e.g., Zika virus [30] and poxviruses [25]) by sequencing PCR amplicons (AmpSeq). The MinION, therefore, represents an opportunity to take infectious disease diagnostics a step further and to perform rapid identification and genetic characterization of infectious agents at a lower cost.

As with any deep sequencing platform, the sequence analysis approach is integral for accurate interpretation. Primarily, two approaches for taxonomic profiling of microbial sequencing data have been employed: read-based and de novo assembly-based classifications. Read-based metagenomic classification software has been used for identification of microbial species from high-throughput sequencing data [23, 31–33]. Although the sequencing accuracy of the MinION is improving, the raw single-read error rate of nearly 10% [34] may limit the accuracy of this approach for Nanopore data [31], especially when attempting to subspecies level differentiation. De novo approaches that use quality-based filtering and clustering of reads [35], or use consensus-based error correction of Nanopore sequencing reads have been reported [36]; however, these are not optimized for amplicon sequencing data.

In this study, a specific and rapid protocol, using the MinION sequencer, was developed to detect representative isolates from all currently circulating (excluding the Madagascar-limited genotype XI) genotypes of NDV. This protocol was also tested on 15 clinical swab samples collected from chickens during disease outbreaks. Additionally, a Galaxy-based, de novo AmpSeq workflow is presented that results in accurate final consensus sequences allowing for accurate genotype and virulence prediction. This study represents the first step towards developing AmpSeq as a diagnostic tool for NDV.

## Methods

### Viruses and clinical samples

Thirty-three NDV isolates, representing 15 different genotypes of different virulence, and ten other avian avulaviruses (AAvV 2–10 and AAvV-13) from the Southeast Poultry Research Laboratory (SEPRL) repository, were propagated in 9–11-day-old specific pathogen free (SPF) eggs [37] and the harvested allantoic fluids were used in this study. Additionally, 15 oral and cloacal swab samples collected from chickens during disease outbreaks in Pakistan in 2015 were collected, and the resulting swab fluid was shipped on dry ice, and then stored at − 80 °C. RNA was extracted as described below for both egg-grown and clinical swab samples. The background information of the egg-grown isolates and the clinical samples is summarized in Additional file 1: Table S1 and Table S2, respectively.

### RNA extraction

Total RNA from each sample was extracted from infectious allantoic fluids or directly from clinical swab media using TRIzol LS (Thermo Fisher Scientific, USA) following the manufacturer’s instructions. RNA concentrations were determined by using Qubit® RNA HS Assay Kit on a Qubit® fluorometer 3.0 (Thermo Fisher Scientific, USA).

### Amplicon synthesis and MinION library preparation

Approximately 20 ng (in 5 μl) of RNA was reverse transcribed, and cDNA was amplified with target-specific primers using the SuperScript™ III One-Step RT-PCR System (Thermo Fisher Scientific, USA). Previously published primers (4331F and 5090R) [9, 38] were used in this protocol to target NDV; however, the primers were tailed with universal adapter sequence of 22 nucleotides (in bold font) to allow PCR-based barcoding: 4331F Tailed: 5′-**TTTCTGTTGGTGCTGATATTGC**GAGGTTACCTCYACYAAGCTRGAGA-3′; 5090R Tailed: 5′-**ACTTGCCTGTCGCTCTATCTTC**TCATTAACAAAYTGCTGCATCTTCCCWAC-3′). The thermocycler conditions for the reaction were as follows: 50 °C for 30 min; 94 °C for 2 min; 40 cycles of 94 °C for 15 s, 56 °C for 30 s, and 68 °C for 60 s, followed by 68 °C for 5 min. The reaction amplified a 788 base pair (bp) NDV product (832 bp including primer tails) for all genotypes, which included 173 bp of the 3′ region of the end of the M gene and 615 bp of the 5′ end of the F gene (sizes and primer locations based on the Genotype V strain). Amplified DNA was purified by Agencourt AMPure XP beads (Beckman Coulter, USA) at 1.6:1 volumetric bead-to-DNA ratio and quantified using the dsDNA High Sensitivity Assay kit on a Qubit® fluorometer 3.0. MinION-compatible DNA libraries were prepared with approximately 1 μg of barcoded DNA in a total volume of 45 μL using nuclease-free water and using the 1D PCR Barcoding Amplicon Kit (Oxford Nanopore Technologies, UK) in conjunction with the Ligation Sequencing Kit 1D (SQK-LSK108) [23] as per manufacturer’s instructions. Briefly, each of the amplicons were diluted to 0.5 nM for barcoding and amplified using LongAmp Taq 2X Master Mix (New England Biolabs, USA) with the following conditions; 95 °C for 3 min; 15 cycles of 95 °C for 15 s; 62 °C for 15 s, 65 °C for 50 s, followed by 65 °C for 50 s. The barcoded amplicons were bead purified, pooled into a single tube, end prepped, dA tailed, bead purified, and ligated to the sequencing adapters per manufacturer’s instructions. Final DNA libraries were bead purified and stored frozen until used for sequencing.

### Comparison of AmpSeq protocol to RT-qPCR assay

For comparison of this MinION-based protocol with the matrix gene reverse transcriptase-quantitative polymerase chain reaction (RT-qPCR) assay [10], both methods were used on a dilution series from a single isolate. NDV (LaSota strain) from the SEPRL repository was cultured in SPF 9–11-days-old eggs and the harvested allantoic fluids were diluted to titers ranging from 10^6^ to 10^1^ EID_50_/mL in brain-heart infusion broth. RNA was extracted from dilutions, and DNA libraries were prepared following the same protocols as described above. Amplicons from each of the dilutions were barcoded separately. At the pooling step, equal concentrations of barcoded amplicons from different dilutions of LaSota were pooled together in single tube. Dilutions, extractions, library construction, and sequencing were performed twice (run 1 and run 2).

The same extracted RNA was also used as the input into the RT-qPCR using the AgPath-ID one-step RT-PCR Kit (Ambion, USA) on the ABI 7500 Fast Real-Time PCR system following the previously described protocols [10].

### Sequencing by MinION

The libraries were sequenced with the MinION Nanopore sequencer [23]. A new FLO-MIN106 R9.4 flow cell, stored at 4 °C prior to use, was allowed to equilibrate to room temperature for 10 min before priming it for sequencing. The flow cell was primed with running buffer as per manufacturer’s instructions. The pooled DNA libraries were prepared by combining 12 μL of the libraries with 2.5 μL nuclease-free water, 35 μL RBF, and 25.5 μL library loading beads. After the MinION Platform QC run, the DNA library was loaded into the MinION flow cell via the SpotON port. The standard 48-h 1D sequencing protocol was initiated using the MinKNOW software v.5.12. Detailed information for all MinION runs in this study is provided in Additional file 1: Table S3.

The complete steps from RNA isolation to MinION sequencing were performed twice for egg-grown viruses. One run consisted of six egg-grown isolates from different genotypes representative of vaccine and virulent NDV strains (run 3: 6-sample pool). The other run consisted of these same six viruses and an additional 27 egg-grown NDV isolates (run 4: 33-sample pool). The clinical samples (*n* = 15) were processed in runs 5, 6, and 7. A variable number of samples were pooled in these three sequencing runs to cluster libraries with similar concentrations.

To determine the accuracy of consensus sequences at different sequencing time points for accurate identification of the NDV genotypes, the raw data (FAST5 files) obtained from the 10-fold serial dilution experiment (see above) were analyzed in subgroups based on time of acquisition and processed through the AmpSeq workflow as described below.

### Development of MinION data analysis workflow

**Fig. 1:**
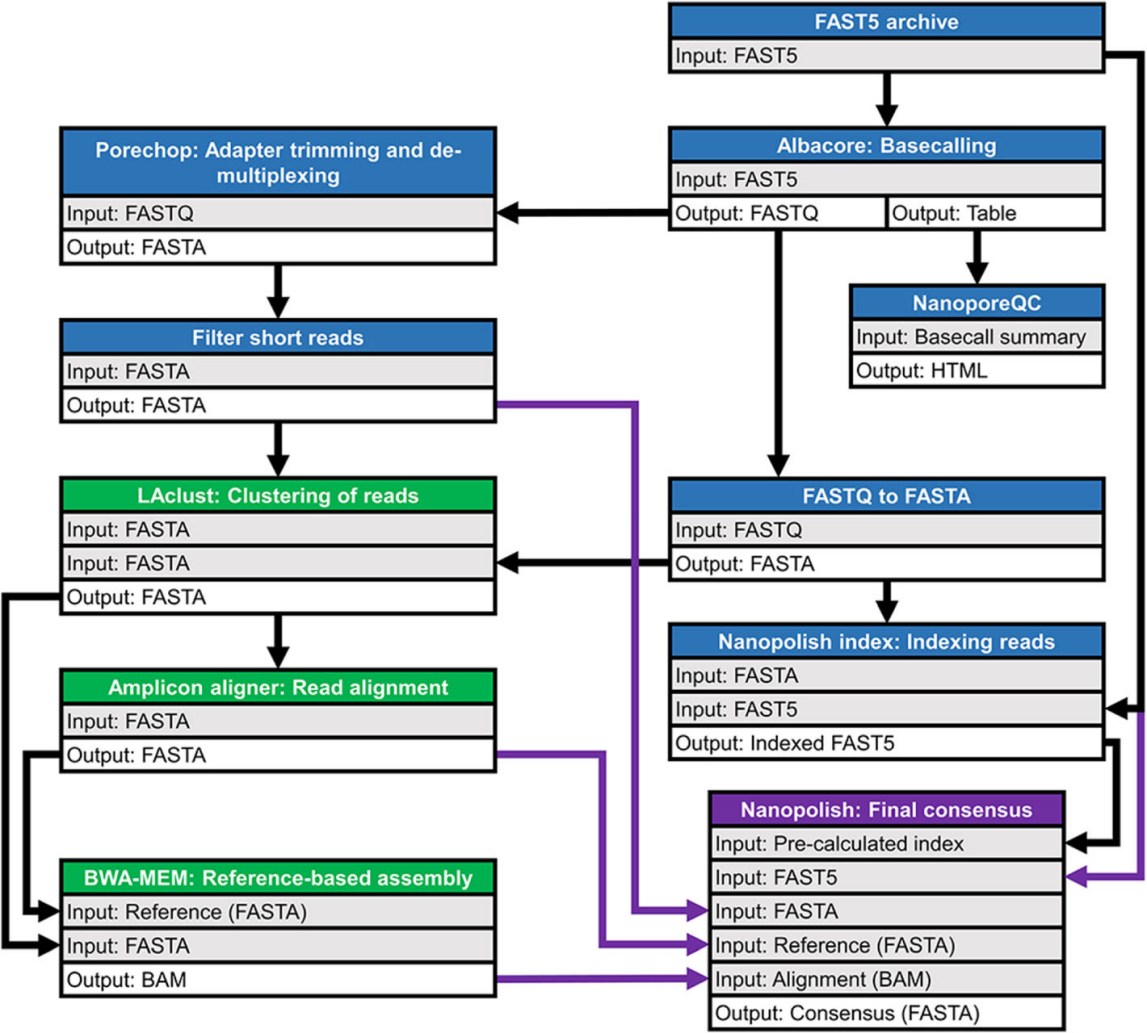
Schematic diagram of customized Galaxy workflow for MinION sequence data analysis. *Blue shading* indicates pre-processing steps. *Green shading* indicates post-processing steps; assembly/output is shaded purple. *Purple* arrows indicate different inputs for final consensus calculation

To analyze the Nanopore sequencing data, a custom, assembly-based AmpSeq workflow within the Galaxy platform interface [39] was developed, as diagrammed in Fig. 1. The MinION raw reads in FAST5 format were archived (tar format) and uploaded into Galaxy workflow. The reads were base-called using the Albacore v2.02 (ONT). The NanoporeQC tool v0.001 (available in the Galaxy testing toolshed) was used to visualize read quality based on the summary table produced by Albacore. Porechop v0.2.2 [40] was used to demultiplex reads for each of the barcodes and trim the adapters at the ends of the reads by using default settings. Short reads (cutoff = 600 bp) were filtered out and the remaining reads were used as input to the in-house LAclust v0.002. LAclust performs single-linkage clustering of noisy reads based on alignment identity and length cutoffs from DALIGNER pairwise alignments [41] (minimum alignment coverage = 0.90, maximum identity difference = 0.35; minimum number of reads to save cluster = 5; maximum reads saved per cluster = 200, minimum read length = 600 bp; rank mode = number of intracluster linkages; randomized input read order = yes). Read clusters generated by LAclust were then aligned using the in-house Amplicon aligner v0.001 to generate a consensus sequence. This tool optionally subsamples reads (target depth used = 100), re-orients them as necessary, aligns them using Multiple Alignment using Fast Fourier Transform (MAFFT) [42] with highly relaxed gap opening and extension penalties, and calls a majority consensus. Next, each consensus was used as a reference sequence for mapping the full unfiltered read clusters from LAclust with BWA-MEM and ONT2D settings [43, 44]. The final consensus sequence for each sample was refined by using Nanopolish v0.8.5 [45], which calculates an improved consensus using the read alignments and raw signal information from the original FAST5 files. After manually trimming primer sequences from both 3′ (25 bp) and 5′ (29 bp) ends, the obtained consensus sequences (734 bp) were BLAST searched against NDV customized database, which consisted NCBI’s nucleotide (nt) database and internal unpublished NDV sequences (NCBI database updated on May 23, 2018).

### Sequencing by MiSeq

For comparison between nucleotide sequences obtained from MinION and MiSeq (a high accuracy sequencing platform), 24 NDV isolates from the SEPRL repository that were used for MinION sequencing (representing each currently circulating genotype except XI and multiple sub-genotypes of NDV) and 15 clinical swab samples (allantoic fluid of cultured swab samples) were processed for target-independent NGS sequencing. Briefly, paired-end random sequencing was conducted from cDNA libraries prepared from total RNA using KAPA Stranded RNA-Seq kit (KAPA Biosystems, USA) as per manufacturer’s instructions and as previously described [46]. All libraries for NGS were loaded into the 300-cycle MiSeq Reagent Kit v2 (Illumina, USA) and pair-end sequencing (2 × 150 bp) was performed on the Illumina MiSeq instrument (Illumina, USA). Pre-processing and de-novo assembly of the raw sequencing data was completed within the Galaxy platform using a previously described approach [19].

### Phylogenetic analysis

The assembled consensus sequences from different NDV genotypes and sub-genotypes (6 sequences from MinION run 3, 33 sequences from run 4 and 24 sequences from MiSeq; a total of 62 sequences) and selected (minimum of one sequence from each genotype/subgenotype) sequences from GenBank (*n* = 66) were aligned using ClustalW [47] in MEGA6 [48]. Determination of the best-fit substitution model was performed using MEGA6, and the goodness-of-fit for each model was measured by corrected Akaike information criterion (AICc) and Bayesian information criterion (BIC) [48]. The final tree was constructed using the maximum-likelihood method based on the General Time Reversible model as implemented in MEGA6, with 500 bootstrap replicates [49]. The available GenBank accession number for each sequence in the phylogenetic tree is followed by the, host name, country of isolation, strain designation, and year of isolation.

### Comparison of MinION and MiSeq sequence accuracy

To assess the accuracy of the MinION AmpSeq consensus sequences, 24 samples were sequenced by both deep-sequencing methods (MinION and MiSeq) described above. Pairwise nucleotide comparison between MinION and MiSeq was conducted using the Maximum Composite Likelihood model [50]. The variation rate among sites was modeled with a gamma distribution (shape parameter = 1). The analysis involved 54 nucleotide sequences. Codon positions included were 1st + 2nd + 3rd + Noncoding. All positions containing gaps and missing data were eliminated. There were a total of 691 positions in the final dataset. The evolutionary distances were inferred by pairwise analysis using the MEGA6 [48].

## Results

### Comparison to the matrix gene RT-qPCR assay

Six, sequential, 10-fold dilutions (from 10^6^ EID_50_/ml to 10^1^ EID_50_/ml) from one NDV isolate (LaSota) were used to compare the ability of AmpSeq and RT-qPCR to detect low quantities of NDV. In each of the six dilutions, AmpSeq and the matrix RT-qPCR detected NDV in all dilutions. AmpSeq resulted in 99.04–100.0% sequence identity to the LaSota isolate across all six dilutions in the first experiment (run 1) and 99.86–100.0% identity in the second experiment (run 2) (Table 1).

**Table 1:** Comparison of MinION sequencing to RT-qPCR for detection of NDV LaSota (runs 1 and 2)

Dilution (EID50/ml)	Total reads^a^	Total NDV reads^b^	Reads per consensus^c^	Percent identity^d^	Consensus length	RT-qPCR^e^ (Ct)
	R1^f^ | R2^g^	R1 | R2	R1 | R2	R1 | R2	R1 | R2	R1 | R2
10^6	6667 | 11,366	6577 | 10,861	200 | 200	100 | 100	734 | 734	21.8, 21.1 | 22.7, 22.7
10^5	4519 | 6801	4439 | 6540	200 | 200	100 | 100	734 | 734	26.3, 25.8 | 26.2, 26.4
10^4	3856 | 8289	3829 | 7890	200 | 200	100 | 100	734 | 734	28.9, 27.8 | 29.1, 29.3
10^3	164 | 9484	157 | 9061	157 | 200	100 | 100	734 | 734	31.4, 31.1 | 32.7, 32.8
10^2	94 | 4939	85 | 4725	85 | 200	100 | 100	734 | 734	34.2, 34.8 | 34.8, 35.3
10^1	133 | 2652	131 | 2520	131 | 200	99.04 | 99.86	729 | 734	34.9, 34.8 | 36.9, 36.7

^**a**^Obtained from output of Porechop

^b^Obtained from output of LAclust

^c^Obtained from output of BWA-MEM. Input into BWA-MEM was limited to 200 reads based on LAclust options

^d^Consensus sequence identity to the reference sequence of NDV LaSota sequenced with MiSeq

(MH392212/chicken/USA/LaSota/1946)

^e^Each dilution performed in duplicate and threshold cycle (Ct) values from each well are shown here

^f^Run 1

^g^Run 2

Note: All 60,000 reads obtained during 32 min of sequencing run (R1) were utilized for the analysis. All 98,916 reads from R2 were utilized for the analysis

### Time for data acquisition and analysis

To determine the minimal sequencing time needed for acquisition of accurate full-length amplicon consensus sequences at different serial dilutions, 28,000 reads, which were obtained within the first 19 min of sequencing in the first serial dilution experiment (run 1), were analyzed. For all concentrations, the first read that aligned to the reference LaSota sequence was obtained within 5 min after the sequencing run started. To obtain consensus sequences (5 reads required to build a consensus sequence) only 5 min of sequencing time were required for concentrations 10^6^–10^3^ EID_50_/ml, which resulted in 99.18–100% sequence identity to the reference LaSota strain. Seven minutes were required to obtain NDV consensus sequences for the two lower concentrations: 10^1^ EID50/ml = 8 reads, 98.77% identity and 10^2^ EID_50_/ml = 5 reads, 98.37% identity (Table 2). After as little as 10 min of sequencing, the identity to the reference sequence was above 99% for even in the most dilute sample.

**Table 2:** Accuracy of consensus sequence from serial dilutions (EID_50_/ml) of NDV LaSota during MinION sequencing run 1

Sequencing run time (min)	Total Raw reads	10^6		10^5		10^4		10^3		10^2		10^1	
		NDV reads^a^	% Identity^b^	NDV reads	% Identity	NDV reads	% Identity	NDV reads	% Identity	NDV reads	% Identity	NDV reads	% Identity
5	4000	283	100	199	100	182	100	10	99.18	2	NA	3	NA
7	8000	644	100	406	100	368	100	14	99.32	8	98.77	5	98.37
10	12,000	1029	100	661	100	571	100	23	99.32	16	99.32	8	99.18
12	16,000	1397	100	901	100	759	100	29	99.86	17	99.32	14	99.45
14	20,000	1772	100	1173	100	1014	100	36	99.73	20	99.45	17	99.59
16	24,000	2183	100	1451	100	1231	100	43	99.73	23	99.45	21	99.59
19	28,000	2643	100	1775	100	1498	100	56	99.86	29	99.73	25	99.45

^a^The numbers represent total number of NDV reads obtained from LAclust. A maximum of 200 reads (optional cut-off value) were used to generate full length consensus sequence. Minimum 5 reads were used as a cut-off to build consensus sequence

^b^Consensus sequence identity to the reference sequence of NDV LaSota sequenced with MiSeq

(MH392212/chicken/USA/LaSota/1946)

Consensus sequences were BLAST searched against NDV custom database

Note: Only 28,000 out of total 60,000 reads were utilized for the analysis

### PCR specificity and range of reactivity for NDV genotypes

To determine the utility of the primers for the currently circulating NDV genotypes and the potential cross-reactivity for other AAvVs, which are relatively nonpathogenic in poultry but can confound diagnosis of NDV [50], total RNA from 43 AAvVs, including 23 AAvV-1 genotypes and sub-genotypes (15 different NDV genotypes, 8 different subgenotypes), as well as AAvV-2–10 and − 13 (*n* = 10) were tested. All AAvV-1 genotypes that are currently circulating globally were amplified with tailed primers; samples 19 and 36 had weak bands of the desired molecular weight (i.e., 832 bp) compared to other lanes. Two bands larger than 800 bp were visible on the electrophoresis of samples #19, #20, #21, #31 and #32 (see Additional file 2: Figure S1 legend for interpretation of this result). All non-AAvV-1 viruses failed to produce bands visible by gel electrophoresis (see Additional file 2: Figure S1).

### Quality metrics

**Fig. 2:**
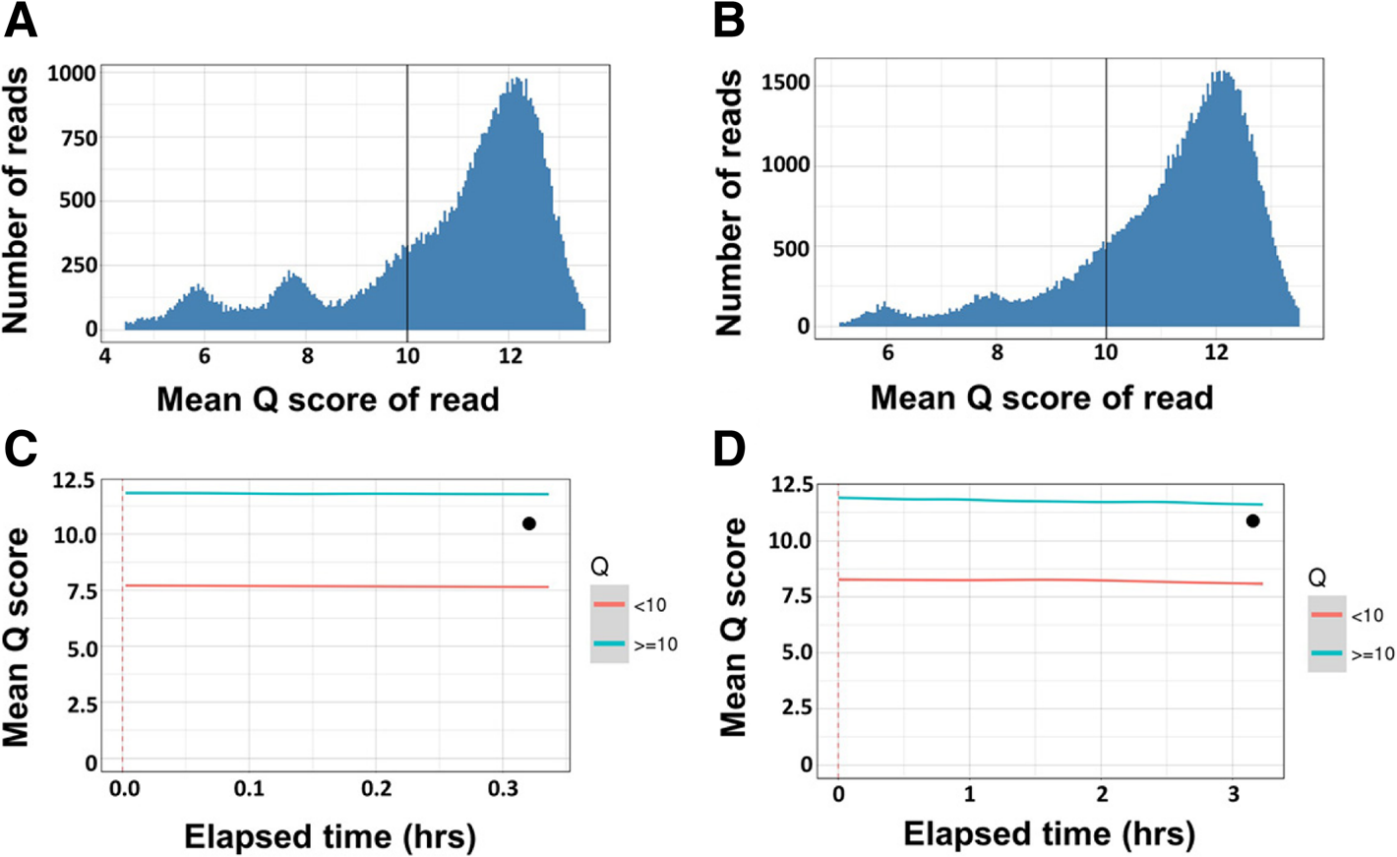
Quality metrics of two MinION sequencing runs. Mean read-based quality score distribution of 6 sample pooled run (run 3) (**a**) and 33 sample pooled run (run 4) (**b**). Mean run-based quality score over time of six sample pooled run (**c**) and 33 sample pooled run (**d**). The overall read quality average (●) remained above 10 in both runs

**Fig. 3:**
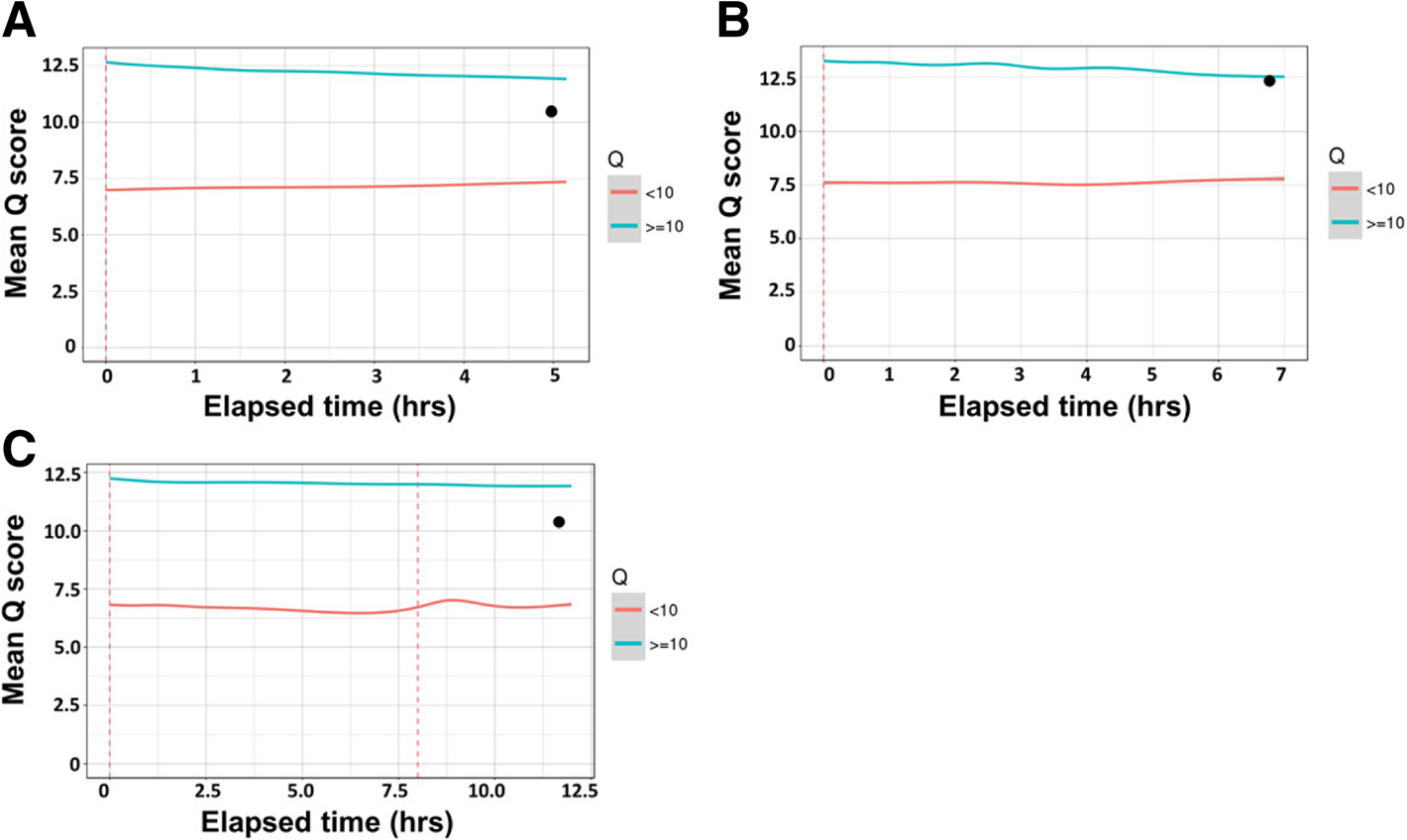
Mean Q _≥10_ (blue lines) and Q _< 10_ (orange lines) of total reads over time during MinION sequencing runs using clinical swab samples. Additionally, the overall read quality average (●) for all three runs, remained above 10. **a**: MinION run 5, *n* = 9 samples, runtime = 6 h. **b**: MinION run 6, *n* = 4 samples, runtime = 7 h. **c**: MinION run 7, *n* = 2 samples, runtime = 12 h

The Nanopore QC tool was used to obtain quality metrics plots of all sequencing runs. For MinION runs 3 (6-sample pool) and 4 (33-sample pool), more than 70% of total reads had a quality score greater than ten (Q10 score = 90% accuracy) (Fig. 2a and b). The average overall mean read quality scores in both runs were comparable (run 3 = 10.7, run 4 = 11.0), and the mean quality scores of reads ≥10 (mean Q_≥10_) were similar (11.8) for both runs (Fig. 2 C and D). In addition, analysis of five consecutive batches of reads (each batch = 20,000 reads) obtained at different time intervals from run 4 indicated that the overall mean read quality for each 20,000 read batch remained above 10 (see Additional file 3: Table S4). Similarly, the mean Q_≥10_ over time remained consistent in the clinical sample runs (runs 5–7), which had long (12 h) sequencing runs (Fig. 3a, b and c, blue lines).

### Sub-genotypic resolution of AAvV-1 viruses with MinION sequencing

To determine the capability to effectively detect and differentiate viruses of different genotypes and sub-genotypes, PCR amplicons from 33 egg-grown isolates, which were representative of 23 different NDV genotypes and sub-genotypes, were barcoded, pooled, and sequenced in a single 12-h MinION run (run = 4) generating a total of 2.076 million reads. The first 100,000 reads, which were obtained in 3 h and 10 min, were analyzed for identification of all 33 NDV isolates used in the study. All 33 NDV isolates were correctly identified to the sub-genotype level (Table 3), with 97.82–100% sequence identity. Thirty-one of thirty-three samples were greater than 99% identical to the expected genotype in each of the sample, with 22 of 33 having 100% sequence identity. Samples with higher sequence identity represent those isolates whose sequences (Sanger or MiSeq based) had already been deposited in GenBank, while those samples with lower sequence identity lacked replicate sequences from those particular isolates. For sample #37, MiSeq detected genotypes XIIIb and VIc, but the AmpSeq workflow only detected genotype XIIIb (e.g., see pairwise comparison section for further demonstration of this protocol’s accuracy).

**Table 3:** Identification and virulence prediction of NDV genotypes from 33 egg-grown samples (runs 3 and 4)

Sample ID	Input genotype^a^	Output genotype^b^	BLAST search	Alignment length	Percent identity	Fusion protein cleavage site^c, □^
**1**	II*	**II**	**MH392212/chicken/USA/LaSota/1946**	**732**	**100**	**low virulent**
**2**	**II**	**II**	**KJ607167/LHLJ/2/goose/2006/China**	**734**	**100**	**low virulent**
**3**	**II**	**II**	**KJ607167/LHLJ/2/goose/2006/China**	**732**	**100**	**low virulent**
**4**	**II**	**II**	**EU289029/turkey/USA/VG/GA-clone_5/1987**	**734**	**99.86**	**low virulent**
**5**	Ia*	**Ia**	**MH392213/chicken/Australia/Queensland/V-4/10/1966**	**734**	**100**	**low virulent**
**15**	**Ia**	**Ia**	**MH392213/chicken/Australia/Queensland/V-4/10/1966**	**734**	**99.86**	**low virulent**
16	VIId	VIId	KU295454/chicken/Ukraine/Lyubotyn/961/2003	735	99.46	virulent
17	II*	II	MH392228/poultry/Canada/Ontario/Berwick/853/1948	735	100	virulent
18	II	II	MH392228/poultry/Canada/Ontario/Berwick/853/1948	732	98.36	virulent
19	III*	III	*MH392214/chicken/India/Mukteswar/519/1940	734	100	virulent
20	IV*	IV	MH392215/chicken/Nigeria/Kano/1973/N52/899/1973	734	99.86	virulent
21	IV	IV	EU293914/Italy/Italien/1944	734	99.05	virulent
22	XIVb*	XIVb	KT948996/domestic_duck/Nigeria/NG-695/KG.LOM.11–16/2009	734	100	virulent
23	Va*	Va	MH392216/cormorant/USA/MN/92–40,140/250/1992	734	100	virulent
24	Vb*	Vb	MH392217/turkey/Belize/4338-4/607/2008	734	100	virulent
25	Vc*	Vc	MH392218/chicken/Mexico/NC/23/686/2011	733	99.73	virulent
26	VIc*	VIc	KY042125/chicken/Bulgaria/Dolno_Linevo/1992	734	100	virulent
27	VIm*	VIm	KX236101/pigeon/Pakistan/Lahore/25A/2015	734	100	virulent
28	VIIj*	VIIj	MH392219/chicken/Egypt/Sohag/18/1020/2014	734	100	virulent
29	VIIe	VIIe	KJ782375/goose/China/GD-QY/1997	734	97.82	virulent
30	VIIi*	VIIi	KX496962/ wild_pigeon/Pakistan/Lahore/20A/996//2015	734	100	virulent
31	IX*	IX	MH392220/poultry/China/04-23/C12/647/2004	734	100	virulent
32	IX	IX	MH392220/poultry/China/04–23/C12/647/2004	734	99.86	virulent
**33**	Xb*	**Xb**	**MH392221/mallard/USA/MN/99–376/163/1999**	**734**	**100**	**low virulent**
**34**	Xa*	**Xa**	**GQ288378/ northern_pintail/USA/OH/87–486/1987**	**734**	**100**	**low virulent**
35	XIIa*	XIIa	JN800306/poultry/Peru/1918-03/2008	734	100	virulent
36	XIIIb*	XIIIb	MH392222/chicken/Pakistan/SPVC/Karachi/27/558/2007	734	99.18	virulent
37^d^	VIc/XIIIb*	XIIIb	MH392223/chicken/Pakistan/SPVC/Karachi/33/556-XIII/2007	734	99.46	virulent
38	XIVb*	XIVb	MH392225/chicken/Nigeria/KD/TW/03 T/N45/720/2009	734	100	virulent
39	XVI*	XVI	MH392226/chicken/Dominican_Republic/FO/499–31/505/2008	734	100	virulent
40	XVIIa*	XVIIa	KY171995/VRD124/06/N11/867/chicken/2006/Nigeria	734	100	virulent
41	XVII*	XVII	KU058680/903/domestic_duck/Nigeria/KUDU-113/1992	734	100	virulent
42	XVIIIb*	XVIIIb	MH392227/chicken/Nigeria/OOT/4/1/N69/914/2009	734	100	virulent

^a^Input genotype was determined with MiSeq sequencing (*) or previous Sanger sequencing

^b^Determined by MinION sequencing

^c^F protein cleavage sites of virulent NDV genotypes contains more than 3 basic amino acids [(112(R/K)-R-(Q/K/R)-(R/K)-R-F117)] and low virulent NDV genotypes has monobasic amino acids [(112(G/E)-(R/K)-Q-(G/E)-R-L117)]

^d^Illumina Miseq detected two NDV genotypes

*Matching MiSeq result from same isolate

^□^ The fusion protein cleavage sites did not vary between AmpSeq and either previous Sanger or previous MiSeq

Note: Isolates known to have low virulence are highlighted in bold

While 832 bp was the expected amplicon size, genotypes III, IV, and IX (all previously untested genotypes with this primer set) yielded an unexpected electrophoresis product of ~ 1000 bp (see above). The analysis of sequences obtained from these NDV isolates revealed that in addition to the 788 bp adapter-trimmed consensus sequence, an upstream region of NDV genome was amplified, resulting in an 1067 bp adapter-trimmed consensus sequence that contains the targeted NDV sequence.

### Clinical swab samples from chicken

To assess the potential utility of this protocol on field samples from disease outbreaks, MinION libraries were generated directly from clinical swab samples. These swab samples were also propagated in eggs and the allantoic fluid was sequenced using a MiSeq-based workflow (runs 5, 6, and 7) to compare to the MinION results. Out of 11 NDV-positive samples with the MiSeq method, 10 samples were NDV positive by the MinION protocol (Sample #52 being the exception) (Table 4). In the six NDV-positive samples that contained one NDV genotype, as detected by the MiSeq method, the same NDV genotype was also detected with the MinION protocol. The MiSeq method detected two genotypes in samples #45, #46, #47, and #49; whereas, the MinION protocol only detected dual genotypes in samples #45 and #46. In sample #48, only one NDV genotype was detected by MiSeq but two NDV genotypes were detected by the MinION protocol. All 4 samples negative by MiSeq were also negative by the MinION protocol.

**Table 4:** Identification and virulence prediction of NDV genotypes in clinical samples collected during outbreaks in 2015 (run 5, 6, and 7)

Sample ID	Miseq genotypes	MinION genotypes	ID of the MinION hit	Reads/ cluster	Consensus length	Percent identity	Fusion protein cleavage site^□^
44	VIIi	VIIi	chicken/Pakistan/Wadana_Kasur/PNI_PF_(14F)/2015	200	734	100	virulent
45	VIIi **II**	VIIi **II**	chicken/Pakistan/Wadana_Kasur/PNI_PF_(14F)/2015 **chicken/USA/LaSota/1946**	28 **5**	734 **733**	99.31 **96.44**	virulent **low virulent**
46	VIIi **II**	VIIi **II**	chicken/Pakistan/Wadana_Kasur/PNI_PF_(14F)/2015 **chicken/USA/LaSota/1946**	10 17	733 733	99.13 **98.51**	virulent **low virulent**
47	VIIi **II**	ND^d^ **II**	NA^e^ **chicken/USA/LaSota/1946**	NA **139**	NA **732**	NA **99.32**	NA **low virulent**
48	**ND** VIIi	**II** VIIi	**chicken/USA/LaSota/1946** chicken/Pakistan/Wadana_Kasur/PNI_PF_(14F)v/2015	**200** 21	**732** 733	**99.59** 99.13	**low virulent** virulent
49	VIIi **II**	ND **II**	NA **chicken/USA/LaSota/1946**	NA **200**	NA **732**	NA **99.32**	NA **low virulent**
50	VIIi	VIIi	chicken/Pakistan/Wadana_Kasur/PNI_PF_(14F)/2015	113	734	100	virulent
51	VIIi	VIIi	chicken/Pakistan/Mirpur_Khas/3EOS/2015	200	734	100	virulent
52^a^	VIIi	ND	NA	NA	NA	NA	NA
53	VIIi	VIIi	exotic Parakeets/Pakistan/Charah/Pk29/29A/2015	5	726	98.5	virulent
54	NO NDV	ND	NA	NA	NA	NA	NA
55	NO NDV	ND	NA	NA	NA	NA	NA
56	NO NDV	ND	NA	NA	NA	NA	NA
57	NO NDV	ND	NA	NA	NA	NA	NA
58	VIIi	VIIi	chicken/Pakistan/Gharoo/Three_star_PF_(7G)/2015	8	729	99.32	virulent
TN^b^	NA	ND	NA	NA	NA	NA	NA
EN^c^	NA	ND	NA	NA	NA	NA	NA

^a^After bead purification, the barcoded amplicon concentration of this sample was lowest in this pool

^b^Template control negative

^c^Negative extraction control

^d^Not detected

^e^Not applicable

^□^ The fusion protein cleavage sites did not vary between AmpSeq and previous MiSeq

Note: Isolates known to have low virulence are highlighted in bold

### Pairwise comparison of replicated MinION sequences and MiSeq sequences

Pairwise nucleotide distance analysis was used to compare the consensus sequences in six samples across two separate MinION runs. There was no variation in the consensus sequence between the MinION runs across those six samples. Pairwise nucleotide distance analysis was also used to compare the MinION consensus sequence to the MiSeq consensus sequence in 24 isolates (one isolate representing each genotype and subgenotype; 24 samples with asterisks in Table 3 were used for pairwise nucleotide comparison). The MinION and MiSeq consensus sequences were 100% identical, except in four samples (#20, #25, #36, and #37), in which the percent identity was 99.18–99.86% (nota bene: the samples in Table 3 without asterisks did not have a second sequence directly from that stock for comparison; thus, the percent identity may be low due to the exact isolate not having a representative sequence in GenBank, e.g., sample 21 with 99.05% similarity). In addition, there were no differences at the fusion gene cleavage site between AmpSeq and either previous Sanger or previous MiSeq results (Tables 3 and 4, last column). Collectively, these results demonstrate the repeatable high accuracy of the MinION-AmpSeq method.

### Phylogeny of NDV genotypes

To confirm the ability of the MinION-acquired partial matrix and fusion gene sequences to be used for accurate analysis of evolutionary relatedness, phylogenetic analysis using consensus sequences (734 bp; trimmed of adapter and primer sequences) obtained from two independent MinION runs (run 3 and 4) was performed. Additionally, the 24 sequences from MiSeq were also included in the phylogenetic tree (Additional file 4: Figure S2, to further illustrate the agreement between these two sequencing methods. In the phylogenetic tree, the isolates (*n* = 33; green font) grouped together with the viruses that showed highest nucleotide sequence identity to them, including those in which MiSeq sequences were available (red font). The six isolates that were sequenced twice (blue font) clustered together. Taken together, the results demonstrated that all sequences clustered to the expected genotype/sub-genotype branch of the phylogenetic tree.

### Time and cost estimation

The time of sample processing and cost estimation of reagents to multiplex and sequence samples (*n* = 6; *n* = 33) from RNA extraction to obtain final consensus sequences is presented in Additional file 3: Table S5. From RNA extraction to final consensus sequence calculations, the average time (including sequencing time) to process six samples was approximately 9–10 person-hours and for 33 samples approximately 26 person-hours. Assuming that flow cell can be used multiple times (twice when 33 samples pooled and five times when six samples pooled to prepare one cDNA library) for sequencing, cost per sequencing run and cost per sample were estimated. The cost per sample decreased from $53 (six samples multiplexed) to $31 (33 samples multiplexed).

## Discussion

This study describes the development of a single protocol for rapid and accurate detection, virulence determination, and preliminary genotype identification (with sub-genotype resolution) of NDV utilizing the low-cost MinION sequencer. Additionally, an assembly-based sequence analysis workflow for MinION amplicon sequencing data was developed. This MinION AmpSeq workflow detected all currently circulating genotypes when using egg-grown viruses. Furthermore, clinical swab samples were used to demonstrate proof of concept that such samples contained sufficient NDV nucleic acid for detection, and interestingly AmpSeq detected 2 NDV genotypes (vaccine and virulent strains) in several clinical swab samples. These capabilities suggest this protocol may be useful for research and ancillary diagnostic procedures and indicates that further development and validation of NDV AmpSeq would be useful, especially in developing countries where NDV is endemic and there is a need for affordable epidemiological surveillance to track reservoirs and disease outbreaks.

The sequence heterogeneity among AAvV-1 genomes, which hinders the ability to develop a single test that sensitively detects NDV while also predicting the genotypic classification and virulence, is well known [4, 51, 52]. Currently, an RT-qPCR targeting the M gene [10] is most sensitive and is used for screening samples, but this assay only provides positive and negative results of the samples. RT-qPCRs that predict virulence based on the fusion gene are available [10, 54]; however, the lower sensitivity of these assays and the inability of at least one of these assays to detect viruses of all genotypes (e.g., genotypes Va and VI) [10, 12, 13] complicate diagnostic interpretation when the matrix and fusion tests have conflicting results. Thus, the only truly reliable option to detect a broad range of viruses and to determine virulence from some strains is to design multiple tests that include genotype-specific primers and probes [7, 12, 53]. Recently, Miller et al. reported that the primer set used in this study detected Class I and all nine of the tested class II genotypes [38]; however, this primer set was not tested against other currently circulating genotypes. The current study includes six additional genotypes, collectively representing all currently circulating genotypes (excluding the Madagascar-limited genotype XI). Furthermore, the ability to use AmpSeq as the final measure of a PCR allows for larger amplicon sizes as compared to RT-qPCR. As such, there will be one less restriction on primer site design when trying to create a pan-NDV primer set. Work is in progress to utilize the ability of MinION to sequence longer amplicon fragments, which will provide more complete phylogenetic information. After optimizing pan-NDV primer design for AmpSeq, sensitivity of pan-NDV AmpSeq will need to be further evaluated.

Additionally, while the preliminary analytical sensitivity of this protocol was determined using only one NDV genotype, the sensitivity of the MinION AmpSeq was comparable to the matrix RT-qPCR test, which does not allow inference of virulence. Further testing of the NDV AmpSeq sensitivity to current virulence-predicting RT-qPCR tests are warranted [14, 15]; however, even these tests lack the genotyping capability of AmpSeq. The ability of this AmpSeq method to detect different genotypes of NDV was further aided by barcoding PCR, which adds another round of PCR to the assay and the ability to adjust the concentration of samples during the library preparation phase. This latter step allows for more volume of low concentration (i.e., weak positives) samples to be added to the library pool. While the additional steps for library synthesis provide these advantages, they also add time to the assay (see below for further discussion of time efficiency). However, the benefit of implementing detection, genotype prediction, and virulence prediction into a single test adds value to this assay.

While the multifaceted nature of this MinION AmpSeq protocol is an advantage, time and cost efficiency must be maintained for it to be useful. MinION is inherently rapid due to the real-time nature of the sequencing. For example, this method identified the correct NDV genotype in all serial dilutions, with an accuracy of 98.37–100%, after only 7 min of sequencing. Because the MinION provides real-time sequence data, it is possible to monitor the sequencing run to determine the optimal run length for each library. Additionally, samples can be multiplexed into a single sequencing run, which reduces time and cost [55]. Recently, multiplexing and MinION sequencing of the PCR products from a panel of 5 samples was reported [55]. Here a panel of 33 samples was multiplexed while maintaining successful NDV genotyping from data collected within 3 h and 10 min of sequencing and without affecting mean read quality and percentage of high-quality reads. Thus, this protocol provides the flexibility to rapidly and economically obtain accurate sequence data for a preliminary genotyping and virulence prediction.

While Nanopore sequencing has numerous benefits, the high error rate poses unique challenges to data analysis. Thus, it is important to extract accurate consensus sequences from raw sequencing data [56]. As previously discussed, pathogen typing from sequencing data can be done with read count-based profiling or de novo assembly approaches [31]. However, there are a limited number of available tools suitable for handling the noisy reads currently produced by the MinION platform. The approach in this study takes advantage of the fact that single MinION reads often represent full-length amplicon sequences. By clustering full-length reads based on pairwise identity and subsequently performing consensus calling using standard multiple alignment software, this method quickly and reliably generates accurate (consistently greater than 99% sequence identity to paired MiSeq) de novo assemblies from amplicon datasets using as few as twenty reads per amplicon, and correct genotypic prediction with as few as five reads per amplicon. Thus, this approach overcomes the inherently high error rate (~ 90% accuracy) of Nanopore sequencing [57] and sequence identification and differentiation at the sub-genotype level can be highly reliable.

One known source of error in Nanopore sequencing is that 5-mers of A and T in the individual reads are difficult to identify accurately with MinION sequencing [58]. Importantly, a 5-mer run of a single base is not present in the cleavage site of the Fusion gene, however, two positions on the consensus sequence where there are 5-mers of A and C are present. Because of 2 instances in which only 4 nucleotides were read (one two nucleotide gap at 153–57 bp position and second on 233 bp) on 5-mer site, sample #37 had less than 100% identity to the respective Miseq data. It should be noted that this type of system error can be easily detected and manually corrected for a paramyxovirus (including NDV), which are viruses that do not tolerate single nucleotide deletions or insertions (rule of six) [59]. Because of the consensus-based approach, despite the relatively high, read-based error rate, the cleavage site was accurately determined.

Because this protocol relies on identity-based clustering prior to assembly, it maintains the ability to detect samples with mixed NDV genotypes. For example, in this study four clinical samples had two different genotypes as detected by MiSeq analysis, two of which were correctly identified by the MinION AmpSeq workflow. In a fifth case, a mixed sample was detected by MinION AmpSeq, but not by MiSeq. A potential explanation for these differences could be that the MiSeq sequencing was performed on egg-amplified samples, which may have altered the relative levels of the two genotypes, as compared to the direct clinical swab sample used for MinION sequencing. Additionally, the differences in molecular techniques (i.e., MinION: targeted; MiSeq: random) may have altered the relative abundance of the genotypes within the sequencing libraries. While further studies into the ability of this workflow to sensitively detect and differentiate NDV in samples with more than one genotype are ongoing, rapid NDV genotyping from clinical samples without culturing the virus in SPF eggs has the potential to facilitate disease diagnostics.

## Conclusions

Taken together, this protocol reliably detected, genotyped, and predicted the virulence of NDV using laboratory stocks of all genetic variants currently circulating worldwide. Furthermore, preliminary testing of clinical-based samples suggests its feasibility using clinical swab samples. This assay can be used for research purposes and as an ancillary test in field investigations; however, further testing, including sensitivity validation on clinical samples and testing the effect of multiple isolates on sensitivity are warranted. Furthermore, the advantages of MinION AmpSeq allow for further optimization not possible with other techniques. For example, PCR product length is less of a restriction with MinION AmpSeq as compared to RT-qPCR. Overall, MinION AmpSeq improves the depth of information obtained from PCRs and allows for more flexibility in assay design, which can be broadly applied to the detection and characterization of numerous infectious agents.

## Additional files

Additional file 1: **Table S1.** The representative genotypes of AAvV-1 and other AAvVs used in this study (egg-grown viruses). **Table S2.** Background information of clinical swab (oral and cloacal) samples collected from chicken during disease outbreaks in Pakistan in 2015. **Table S3.** Detail of MinION sequencing runs. (DOCX 35 kb)

Additional file 2: **Figure S1.** Agarose gel electrophoresis of AAvVs. Samples 6–14 and 43 are AAvVs other than AAvV-1. A DNA ladder (100 bp) was loaded into lane L. A no-template control was loaded into lane N. Bright bands show the amplified target region of AAvA-1 genome (expected product size 832 bp). See Table S1 for key to lanes. An unexpected product of approximately 1100 bp was identified in samples #19, #20, #21, #31, and #32. Analysis of these genomes identified a second potential primer binding site. Additionally, two consensus sequences were obtained from these samples. One was the expected amplicon, and the second was a 1067 bp sequence that corresponded to the predicted second primer binding site (data not shown) and included the targeted amplicon sequence. (DOCX 840 kb)

Additional file 3: **Table S4.** Time-based quality metrics of MinION sequencing run 4 (*n* = 33)**. Table S5.** Estimation of cost of reagents and sample processing time for MinION sequencing. (DOCX 25 kb)

Additional file 4: **Figure S2.** Phylogenetic tree constructed by using the nucleotide sequence (734 bp) of NDV isolates sequenced with MinION and MiSeq, with sequences of related NDV genotypes from GenBank. The evolutionary histories were inferred by using the maximum-likelihood method based on General Time Reversible model with 500 bootstrap replicates as implemented in MEGA 6. The tree with the highest log likelihood (− 9347.8021) is shown. A discrete Gamma distribution was used to model evolutionary rate differences among sites (4 categories [+G, parameter = 0.9254]). The percentages of trees in which the associated sequences clustered together are shown below the branches. The tree is drawn to scale, with branch lengths measured in the number of substitutions per site. The analyses involved 129 nucleotide sequences with a total of 725 positions in the final datasets. The sequences obtained in the current study are denoted with solid circles in front of the taxa name and bold font. Blue circles indicate isolates from MinION sequencing run 1, green circles indicate isolates from MinION sequencing run 2 and red circles indicate MiSeq sequencing. (PPTX 81 kb)

## Abbreviations

AAvV: Avian avulavirus
AICc: Akaike information criterion
AmpSeq: Amplicon sequencing
APMV-1: Avian paramyxovirus 1
BIC: Bayesian information criterion
EID50: 50% egg infectious dose
MAFFT: Multiple Alignment using Fast Fourier Transform
ND: Newcastle disease
NDV: Newcastle disease viruses
ONT: Oxford Nanopore Technologies
RT-qPCR: Reverse transcriptase-quantitative polymerase chain reaction
SEPRL: Southeast Poultry Research Laboratory
SPF: Specific pathogen free
