# Supplementary material for: Rapid virulence prediction and identification of Newcastle disease virus genotypes using third-generation sequencing
Source: Virol J. 2018 Nov 22;15:179. doi: 10.1186/s12985-018-1077-5 (PMC6251111; doi:10.1186/s12985-018-1077-5)
Supplement: Supplementary file 1 — Table S1. The representative genotypes of AAvV-1 and other AAvVs used in this study (egg-grown viruses). Table S2. Background information of clinical swab (oral and cloacal) samples collected from chicken during disease outbreaks in Pakistan in 2015. Table S3. Detail of MinION sequencing runs. (DOCX 35 kb) [file 12985_2018_1077_MOESM1_ESM.docx]

**Table S1.** The representative genotypes of AAvV-1 and other AAvVs used in this study (egg-grown viruses)

| **Sample ID** | **Isolate** | **Genotype/serotype** | **MinION run^a^** |
| --- | --- | --- | --- |
| 1 | chicken/USA/Lasota/1946 | II | 3, 4, MiSeq |
| 2 | chicken/USA/Hitchner/B1/1947 | II | 4 |
| 3 | clone 30 | II | 4 |
| 4 | turkey/USA/VG/GA/1989 | II | 4 |
| 5 | chicken/Australia/Queensland/V-4/10/1966 | Ia | 3, 4, MiSeq |
| 6 | MN2000-495 | APMV-2 | NA |
| 7 | APMV-3/turkey/USA/WI/1968 | APMV-3 | NA |
| 8 | APMV-4/--/USA/MN/2000 | APMV-4 | NA |
| 9 | APMV-6/--/USA/MN/1999 | APMV-6 | NA |
| 10 | APMV-7/--/USA/TX02-12/ | APMV-7 | NA |
| 11 | APMV-8/goose/US/DE/1053/1976 | APMV-8 | NA |
| 12 | APMV-9/duck/USA/NY/22/1978 | APMV-9 | NA |
| 13 | APMV-10/penguin/Falkland Islands/324/2007 | APMV-10 | NA |
| 14 | APMV-13/white-fronted goose/Ukraine/Askania-Nova/48-15-02/2011 | APMV-13 | NA |
| 15 | Malaysia/5091/633/2009 | Ia | 4 |
| 16 | APMV-p-S-221111/964 | VIId | 4 |
| 17 | poultry/Canada/Ontario/Berwick/853/1948 | II | 4, MiSeq |
| 18 | poultry/USA/OH/Miller/778/1948 | II | 4 |
| 19 | chicken/India/Mukteswar/519/1940s | III | 4, MiSeq |
| 20 | chicken/Nigeria/Kano/1973/N52/899/1973 | IV | 4, MiSeq |
| 21 | Italy/Milano/1945 | IV | 4 |
| 22 | duck/Nigeria/NG-695/KG.LOM.11-16/2009 | XIVb | 4, MiSeq |
| 23 | cormorant/USA/MN/92-40140/250/1992 | Va | 4, MiSeq |
| 24 | turkey/Belize/4338-4/607/2008 | Vb | 3, 4, MiSeq |
| 25 | chicken/Mexico/NC/23/686/2011 | Vc | 4, MiSeq |
| 26 | chicken/Bulgaria/Dolno_Linevo/1160/1992 | VIc | 4, MiSeq |
| 27 | pigeon/Pakistan/Lahore/25A/1011/2015 | VIk | 3, 4, MiSeq |
| 28 | chicken/Egypt/Sohag/18/1020/2014 | VIIj | 4, MiSeq |
| 29 | duck/Vietnam/Long Bien/78/2002 | VIIe | 4 |
| 30 | pigeon/Pakistan/Lahore/20A/996/2015 | VIIi | 3, 4, MiSeq |
| 31 | poultry/China/04-23/C12/647/2004 | IX | 4, MiSeq |
| 32 | chicken/03-45/641/2003 | IX | 4 |
| 33 | mallard/USA/MN/99-376/163/1999 | Xb | 4, MiSeq |
| 34 | northern_pintail/US(OH)/87-486/164/1987 | Xa | 4, MiSeq |
| 35 | poultry/Peru/1918-03/603/2008 | XIIa | 4, MiSeq |
| 36 | chicken/Pakistan/SPVC/Karachi/27/558/2007 | XIIIb | 4, MiSeq |
| 37 | chicken/Pakistan/SPVC/Karachi/33/556/2007 | XIIIb, VIc | 3, 4, MiSeq |
| 38 | pigeon/Nigeira/Katsina/KT/MSH/15C_(N2)/689/2009 | XIVb | 4, MiSeq |
| 39 | chicken/Dominican_Republic/FO/499-31/505/2008 | XVI | 4, MiSeq |
| 40 | chicken/Nigeria/VRD124/06/N11/867/2006 | XVIIa | 4, MiSeq |
| 41 | duck/Nigeria/KUDU-113/903/1992 | XVII | 4, MiSeq |
| 42 | chicken/Nigeria/OOT/4/1/N69/914/2009 | XVIIIb | 4, MiSeq |
| 43 | APMV-5/Japan/Tokyo/Kunitachi/1978 | APMV-5 | NA |

^a^ 3 = MinION run 3 (6 samples pooled), 4 = MinION run 4 (33 samples pooled), MiSeq = Isolates sequences with Illumina Miseq. For further information about MinION sequencing runs, see Table S3.

**Table S2.** Background information of clinical swab (oral and cloacal) samples collected from chicken during disease outbreaks in Pakistan in 2015

| **Sample ID** | **Isolate** | **Genotype^a^** | **Swab type** | **MinION run^b^** |
| --- | --- | --- | --- | --- |
| 44 | chicken/Pakistan/Punjab/1F/1062/2015 | VII i | Oral | 6 |
| 45 | chicken/Pakistan/Punjab/2F//1063/2015 | VII i and II | Oral | 5 |
| 46 | chicken/Pakistan/Punjab/4F/1065/2015 | VII i and II | Oral | 6 |
| 47 | chicken/Pakistan/Punjab/5F/1066/2015 | VII i and II | Oral | 5 |
| 48 | chicken/Pakistan/Punjab/7F/1067/2015 | VII i | Oral | 6 |
| 49 | chicken/Pakistan/Punjab/8F/1068/2015 | VII i and II | Oral | 5 |
| 50 | chicken/Pakista/Punjab/14F/1069/2015 | VII i | Cloacal | 5 |
| 51 | chicken/Pakistan/Sindh/4E/1061/2015 | VII i | Oral | 5 |
| 52 | chicken/Pakistan/Punjab/1H/1072/2015 | VII i | Oral | 7 |
| 53 | chicken/Pakistan/Punjab/3H/1074/2015 | VII i | Oral | 5 |
| 54 | chicken/Pakistan/Punjab/16H/1077/2015 | NO NDV | Cloacal | 5 |
| 55 | chicken/Pakistan/Sindh/A3/1221/2015 | NO NDV | Oral | 6 |
| 56 | chicken/Pakistan/Sindh/B1/1222/2015 | NO NDV | Oral | 5 |
| 57 | chicken/Pakistan/Punjab/D2/1226/2015 | NO NDV | Oral | 5 |
| 58 | chicken/Pakistan/Punjab/7G/1071/2015 | VII i | Oral | 7 |

^a^ all isolates were identified as members of AAvV-1 with next-generations sequencing (MiSeq)

^b^ 5 = MinION run 5, 6 = MinION run 6, 7 = MinION run 7. For further information about MinION sequencing runs, see Table S3.

**Table S3.** Detail of MinION sequencing runs

| **Run** | **Description of MinION sequencing run** | **Sample type** | **Sequencing run time** | **Total reads** |
| --- | --- | --- | --- | --- |
| 1 | LaSota serial dilution  (n = 6) (R1) | Allantoic fluid | 32 min | 60,000 |
| 2 | LaSota serial dilutions  (n = 6) (R2) | Allantoic fluid | 32 min | 98916 |
| 3 | NDV6; (n = 6) | Allantoic fluid | 20 min | 60,000 |
| 4 | NDV33; (n = 33) | Allantoic fluid | 12 hrs | 2,084,000 |
| 5 | *Clinical swab samples (n = 9) | Swab material | 6 hrs | 368,000 |
| 6 | *Clinical swab samples (n = 4) | Swab material | 7 hrs | 224,000 |
| 7 | *Clinical swab samples (n = 2) | Swab material | 12 hrs | 284,000 |

*Amplicons obtained from clinical samples were pooled together based on the variation in their concentration
