# Supplementary figures and images for: Rapid virulence prediction and identification of Newcastle disease virus genotypes using third-generation sequencing
Source: Virol J. 2018 Nov 22;15:179. doi: 10.1186/s12985-018-1077-5 (PMC6251111; doi:10.1186/s12985-018-1077-5)

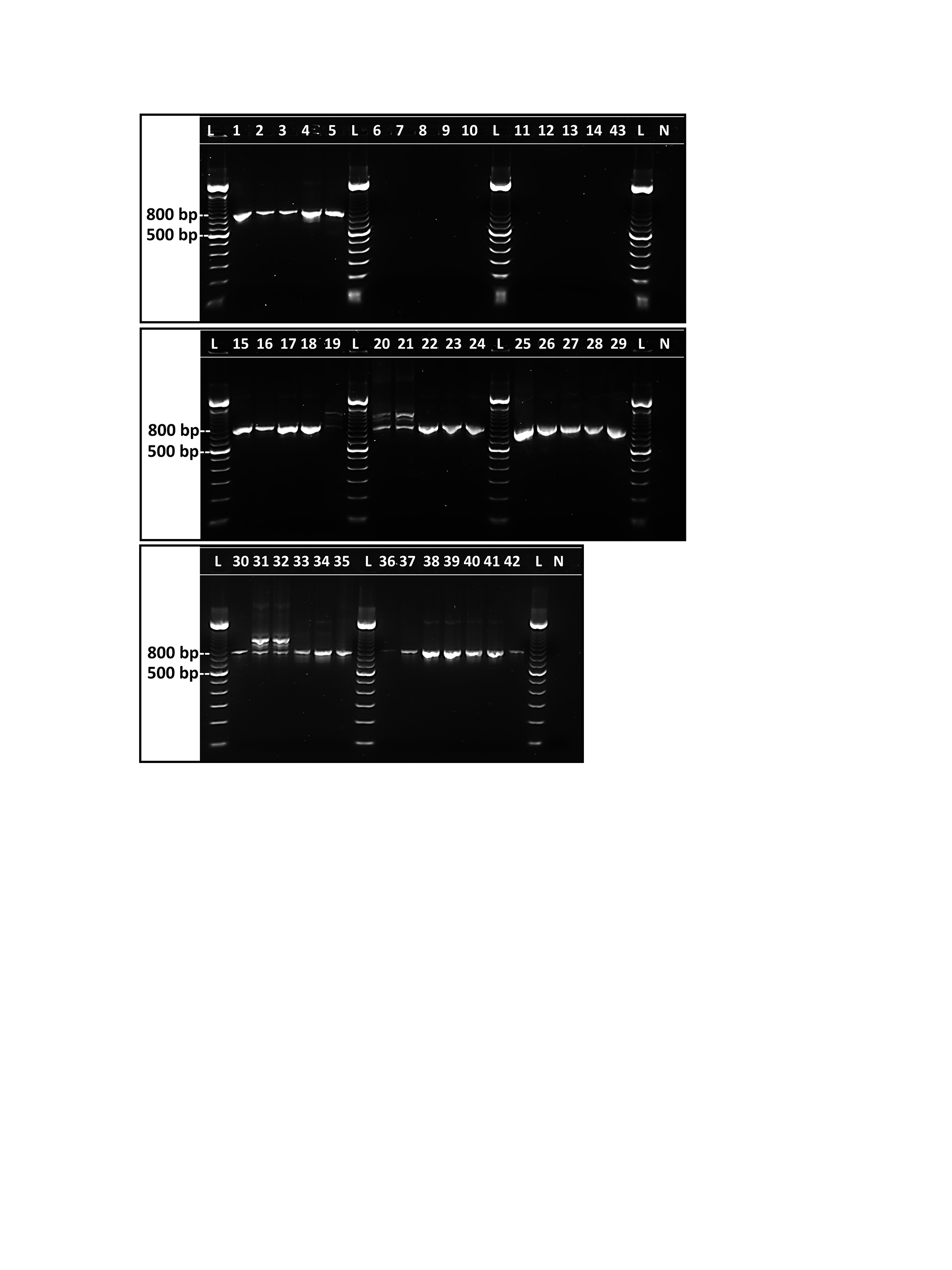

Supplement: Supplementary file 2 — Figure S1. Agarose gel electrophoresis of AAvVs. Samples 6–14 and 43 are AAvVs other than AAvV-1. A DNA ladder (100 bp) was loaded into lane L. A no-template control was loaded into lane N. Bright bands show the amplified target region of AAvA-1 genome (expected product size 832 bp). See Table S1 for key to lanes. An unexpected product of approximately 1100 bp was identified in samples #19, #20, #21, #31, and #32. Analysis of these genomes identified a second potential primer binding site. Additionally, two consensus sequences were obtained from these samples. One was the expected amplicon, and the second was a 1067 bp sequence that corresponded to the predicted second primer binding site (data not shown) and included the targeted amplicon sequence. (DOCX 840 kb) [file 12985_2018_1077_MOESM2_ESM.docx]
