# Supplementary material for: Rapid virulence prediction and identification of Newcastle disease virus genotypes using third-generation sequencing
Source: Virol J. 2018 Nov 22;15:179. doi: 10.1186/s12985-018-1077-5 (PMC6251111; doi:10.1186/s12985-018-1077-5)
Supplement: Supplementary file 3 — Table S4. Time-based quality metrics of MinION sequencing run 4 (n = 33). Table S5. Estimation of cost of reagents and sample processing time for MinION sequencing. (DOCX 25 kb) [file 12985_2018_1077_MOESM3_ESM.docx]

**Table S4.** Time-based quality metrics of MinION sequencing run 4 (n = 33)

| **Sequencing run time for each batch of 20,000 reads (total run time)** | **Mean read quality** | **Reads**  **Q ≥ 10** | **% of reads**  **Q ≥ 10** | **Mean read**  **Q_≥10_** |
| --- | --- | --- | --- | --- |
| 30 (30) min | 11.3 | 16593 | 82.97 | 11.9 |
| 30 (60) min | 11.2 | 16274 | 81.37 | 11.8 |
| 45 (105) min | 11.0 | 15853 | 79.26 | 11.8 |
| 45 (150) min | 11.0 | 15714 | 78.57 | 11.7 |
| 40 (190) min | 10.8 | 15256 | 76.28 | 11.6 |

A Q score of 10 translates into 90% accuracy.

**Table S5.** Estimation of cost of reagents and sample processing time for MinION sequencing

| **Steps** | **Time** | | **Cost in USD** | |
| --- | --- | --- | --- | --- |
| *Multiplexed samples* | *n = 6* | *n = 33* | *n = 6* | *n = 33* |
| RNA extraction | 2 hrs | 8 hrs | 30 | 165 |
| One step RT-PCR | 2 hrs | 2 hrs | 48 | 264 |
| Amplicon purification | 20 min | 2 hrs | 18 | 99 |
| Barcode kit | NA | NA | 25 | 150 |
| Single library preparation | 4 hrs | 8 hrs | 99 | 99 |
| Flow cell | NA | NA | 500 | 500 |
| Flow cell per sequencing run (n) | NA | NA | (n = 5) 100 | (n = 2) 250 |
| Sequencing run | 32 min | 3 hrs 10 min | 320 | 1027 |
| ^a^Basecalling | 1 hr | 1 hr 40 min | NA | NA |
| Post basecalling data processing and consensus assembly | 25 min | 40 min | NA | NA |
| **Total time** | **^b^9-10 hrs** | **26 hrs** | NA | NA |
| **Cost ($) per sample** | NA | NA | **$53** | **$31** |

^a^Basecalling time varies based on average length and total number of reads. For amplicon sizes in this study, basecalling took approximately 60 minutes for 60,000 reads. Longer times will be required for longer amplicons.

^b^Including the sequencing run time
