# Supplementary material for: Rapid virulence prediction and identification of Newcastle disease virus genotypes using third-generation sequencing
Source: Virol J. 2018 Nov 22;15:179. doi: 10.1186/s12985-018-1077-5 (PMC6251111; doi:10.1186/s12985-018-1077-5)
Supplement: Supplementary file 4 — Figure S2. Phylogenetic tree constructed by using the nucleotide sequence (734 bp) of NDV isolates sequenced with MinION and MiSeq, with sequences of related NDV genotypes from GenBank. The evolutionary histories were inferred by using the maximum-likelihood method based on General Time Reversible model with 500 bootstrap replicates as implemented in MEGA 6. The tree with the highest log likelihood (− 9347.8021) is shown. A discrete Gamma distribution was used to model evolutionary rate differences among sites (4 categories [+G, parameter = 0.9254]). The percentages of trees in which the associated sequences clustered together are shown below the branches. The tree is drawn to scale, with branch lengths measured in the number of substitutions per site. The analyses involved 129 nucleotide sequences with a total of 725 positions in the final datasets. The sequences obtained in the current study are denoted with solid circles in front of the taxa name and bold font. Blue circles indicate isolates from MinION sequencing run 1, green circles indicate isolates from MinION sequencing run 2 and red circles indicate MiSeq sequencing. (PPTX 81 kb) [file 12985_2018_1077_MOESM4_ESM.pptx]

## Slide 1
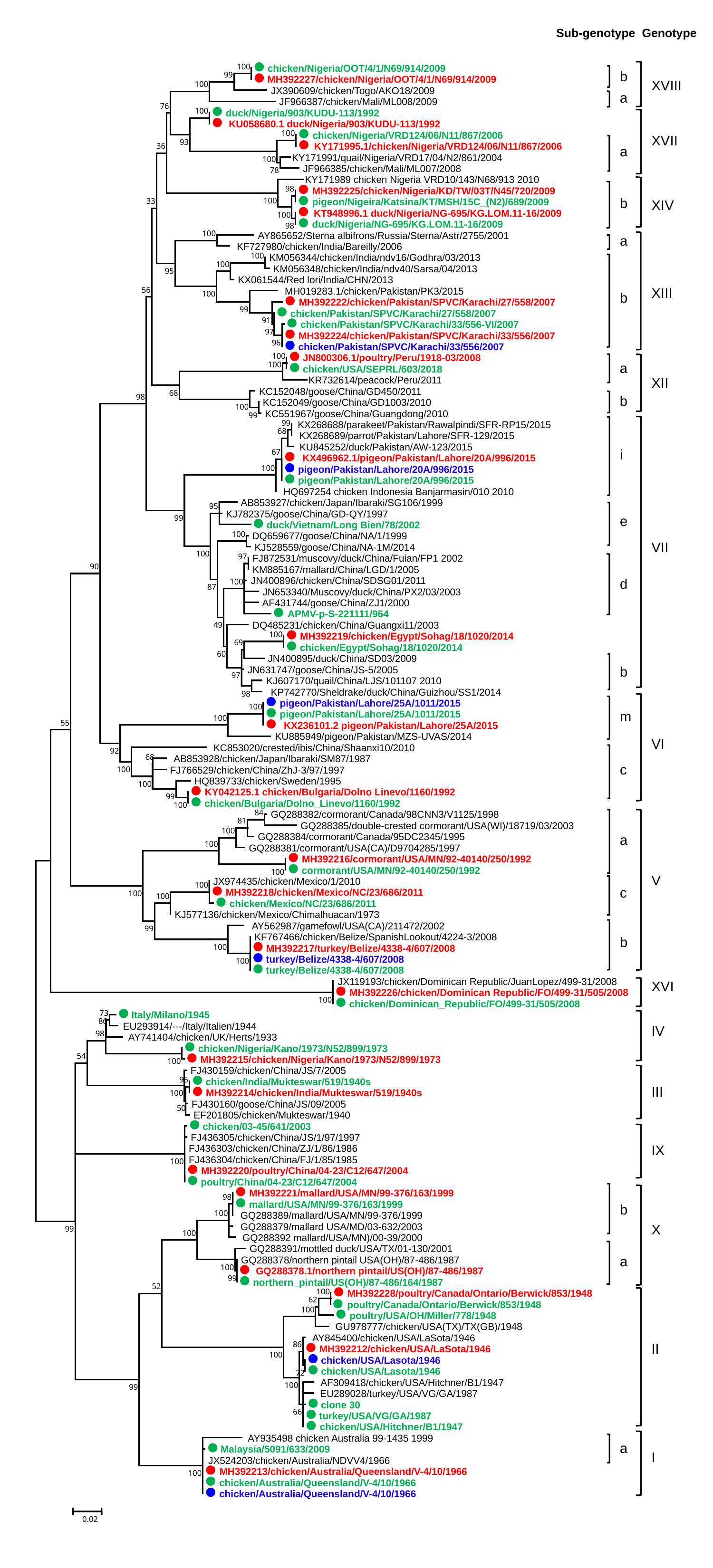

Sub-genotype Genotype
100
 chicken/Nigeria/OOT/4/1/N69/914/2009
 MH392227/chicken/Nigeria/OOT/4/1/N69/914/2009
 JX390609/chicken/Togo/AKO18/2009
 JF966387/chicken/Mali/ML008/2009
 duck/Nigeria/903/KUDU-113/1992
 KU058680.1 duck/Nigeria/903/KUDU-113/1992
 chicken/Nigeria/VRD124/06/N11/867/2006
 KY171995.1/chicken/Nigeria/VRD124/06/N11/867/2006
KY171991/quail/Nigeria/VRD17/04/N2/861/2004
 JF966385/chicken/Mali/ML007/2008
KY171989 chicken Nigeria VRD10/143/N68/913 2010
 MH392225/chicken/Nigeria/KD/TW/03T/N45/720/2009
 pigeon/Nigeira/Katsina/KT/MSH/15C_(N2)/689/2009
 KT948996.1 duck/Nigeria/NG-695/KG.LOM.11-16/2009
 duck/Nigeria/NG-695/KG.LOM.11-16/2009
 AY865652/Sterna albifrons/Russia/Sterna/Astr/2755/2001
 KF727980/chicken/India/Bareilly/2006
 KM056344/chicken/India/ndv16/Godhra/03/2013
 KM056348/chicken/India/ndv40/Sarsa/04/2013
 KX061544/Red lori/India/CHN/2013
 MH019283.1/chicken/Pakistan/PK3/2015
 MH392222/chicken/Pakistan/SPVC/Karachi/27/558/2007
 chicken/Pakistan/SPVC/Karachi/27/558/2007
 chicken/Pakistan/SPVC/Karachi/33/556-VI/2007
 MH392224/chicken/Pakistan/SPVC/Karachi/33/556/2007
 chicken/Pakistan/SPVC/Karachi/33/556/2007
 JN800306.1/poultry/Peru/1918-03/2008
 chicken/USA/SEPRL/603/2018
KR732614/peacock/Peru/2011
 KC152048/goose/China/GD450/2011
 KC152049/goose/China/GD1003/2010
 KC551967/goose/China/Guangdong/2010
 KX268688/parakeet/Pakistan/Rawalpindi/SFR-RP15/2015
 KX268689/parrot/Pakistan/Lahore/SFR-129/2015
 KU845252/duck/Pakistan/AW-123/2015
 KX496962.1/pigeon/Pakistan/Lahore/20A/996/2015
 pigeon/Pakistan/Lahore/20A/996/2015
 pigeon/Pakistan/Lahore/20A/996/2015
 HQ697254 chicken Indonesia Banjarmasin/010 2010
 AB853927/chicken/Japan/Ibaraki/SG106/1999
 KJ782375/goose/China/GD-QY/1997
 duck/Vietnam/Long Bien/78/2002
 DQ659677/goose/China/NA/1/1999
 KJ528559/goose/China/NA-1M/2014
 FJ872531/muscovy/duck/China/Fuian/FP1 2002
 KM885167/mallard/China/LGD/1/2005
 JN400896/chicken/China/SDSG01/2011
 JN653340/Muscovy/duck/China/PX2/03/2003
 AF431744/goose/China/ZJ1/2000
 APMV-p-S-221111/964
 DQ485231/chicken/China/Guangxi11/2003
 MH392219/chicken/Egypt/Sohag/18/1020/2014
 chicken/Egypt/Sohag/18/1020/2014
 JN400895/duck/China/SD03/2009
 JN631747/goose/China/JS-5/2005
 KJ607170/quail/China/LJS/101107 2010
 KP742770/Sheldrake/duck/China/Guizhou/SS1/2014
 pigeon/Pakistan/Lahore/25A/1011/2015
 pigeon/Pakistan/Lahore/25A/1011/2015
 KX236101.2 pigeon/Pakistan/Lahore/25A/2015
 KU885949/pigeon/Pakistan/MZS-UVAS/2014
 KC853020/crested/ibis/China/Shaanxi10/2010
 AB853928/chicken/Japan/Ibaraki/SM87/1987
 FJ766529/chicken/China/ZhJ-3/97/1997
 HQ839733/chicken/Sweden/1995
 KY042125.1 chicken/Bulgaria/Dolno Linevo/1160/1992
 chicken/Bulgaria/Dolno_Linevo/1160/1992
 GQ288382/cormorant/Canada/98CNN3/V1125/1998
 GQ288385/double-crested cormorant/USA(WI)/18719/03/2003
 GQ288384/cormorant/Canada/95DC2345/1995
 GQ288381/cormorant/USA(CA)/D9704285/1997
 MH392216/cormorant/USA/MN/92-40140/250/1992
 cormorant/USA/MN/92-40140/250/1992
 JX974435/chicken/Mexico/1/2010
 MH392218/chicken/Mexico/NC/23/686/2011
 chicken/Mexico/NC/23/686/2011
 KJ577136/chicken/Mexico/Chimalhuacan/1973
 AY562987/gamefowl/USA(CA)/211472/2002
 KF767466/chicken/Belize/SpanishLookout/4224-3/2008
 MH392217/turkey/Belize/4338-4/607/2008
 turkey/Belize/4338-4/607/2008
 turkey/Belize/4338-4/607/2008
 JX119193/chicken/Dominican Republic/JuanLopez/499-31/2008
 MH392226/chicken/Dominican Republic/FO/499-31/505/2008
 chicken/Dominican_Republic/FO/499-31/505/2008
 Italy/Milano/1945
 EU293914/---/Italy/Italien/1944
 AY741404/chicken/UK/Herts/1933
 chicken/Nigeria/Kano/1973/N52/899/1973
 MH392215/chicken/Nigeria/Kano/1973/N52/899/1973
 FJ430159/chicken/China/JS/7/2005
 chicken/India/Mukteswar/519/1940s
 MH392214/chicken/India/Mukteswar/519/1940s
 FJ430160/goose/China/JS/09/2005
 EF201805/chicken/Mukteswar/1940
 chicken/03-45/641/2003
 FJ436305/chicken/China/JS/1/97/1997
 FJ436303/chicken/China/ZJ/1/86/1986
 FJ436304/chicken/China/FJ/1/85/1985
 MH392220/poultry/China/04-23/C12/647/2004
 poultry/China/04-23/C12/647/2004
 MH392221/mallard/USA/MN/99-376/163/1999
 mallard/USA/MN/99-376/163/1999
 GQ288389/mallard/USA/MN/99-376/1999
 GQ288379/mallard USA/MD/03-632/2003
 GQ288392 mallard/USA/MN)/00-39/2000
 GQ288391/mottled duck/USA/TX/01-130/2001
 GQ288378/northern pintail USA(OH)/87-486/1987
 GQ288378.1/northern pintail/US(OH)/87-486/1987
 northern_pintail/US(OH)/87-486/164/1987
 MH392228/poultry/Canada/Ontario/Berwick/853/1948
 poultry/Canada/Ontario/Berwick/853/1948
 poultry/USA/OH/Miller/778/1948
 GU978777/chicken/USA(TX)/TX(GB)/1948
 AY845400/chicken/USA/LaSota/1946
 MH392212/chicken/USA/LaSota/1946
 chicken/USA/Lasota/1946
 chicken/USA/Lasota/1946
 AF309418/chicken/USA/Hitchner/B1/1947
 EU289028/turkey/USA/VG/GA/1987
 clone 30
 turkey/USA/VG/GA/1987
 chicken/USA/Hitchner/B1/1947
 AY935498 chicken Australia 99-1435 1999
 Malaysia/5091/633/2009
 JX524203/chicken/Australia/NDVV4/1966
 MH392213/chicken/Australia/Queensland/V-4/10/1966
 chicken/Australia/Queensland/V-4/10/1966
 chicken/Australia/Queensland/V-4/10/1966
99
100
76
100
100
93
36
100
78
98
100
33
100
98
100
100
100
95
56
100
99
91
97
96
100
100
68
98
100
99
99
68
67
100
95
99
100
100
97
90
100
87
49
100
69
60
97
98
100
100
55
92
68
100
100
99
100
84
81
100
100
100
100
100
100
99
100
100
100
73
86
98
54
100
95
100
50
100
98
100
99
100
100
99
52
100
62
100
86
100
72
100
99
66
100
0.02
b
XVIII
a
XVII
a
b
XIV
a
XIII
b
a
XII
b
i
e
VII
d
b
m
VI
c
a
V
c
b
XVI
IV
III
IX
b
X
a
II
a
I
